# Supplementary figures and images for: Assessment of hospitalization costs and its determinants in infants with clinical severe infection at a public tertiary hospital in Nepal
Source: PLoS One. 2021 Nov 29;16(11):e0260127. doi: 10.1371/journal.pone.0260127 (PMC8629207; doi:10.1371/journal.pone.0260127)

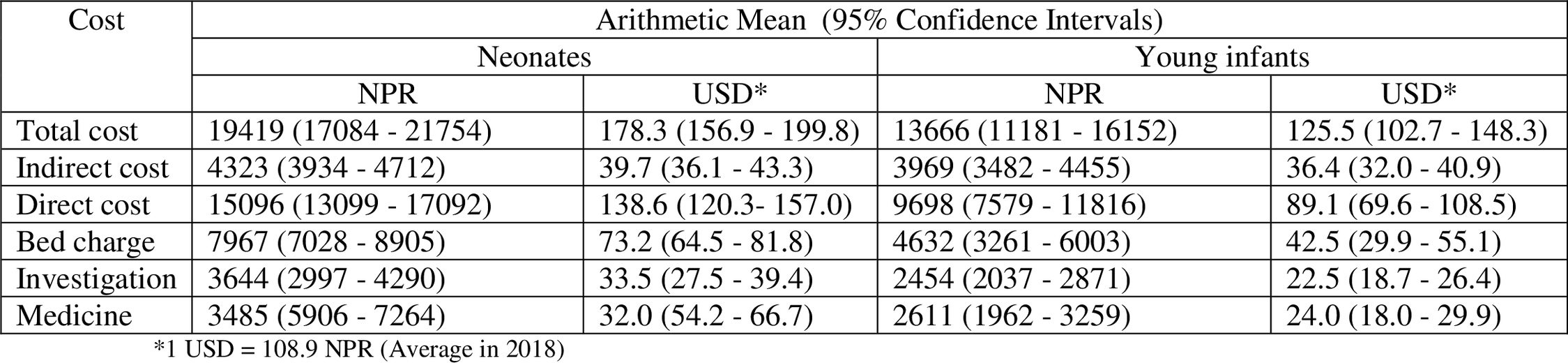

Supplement: S1 Table — (TIF) [file pone.0260127.s001.tif]

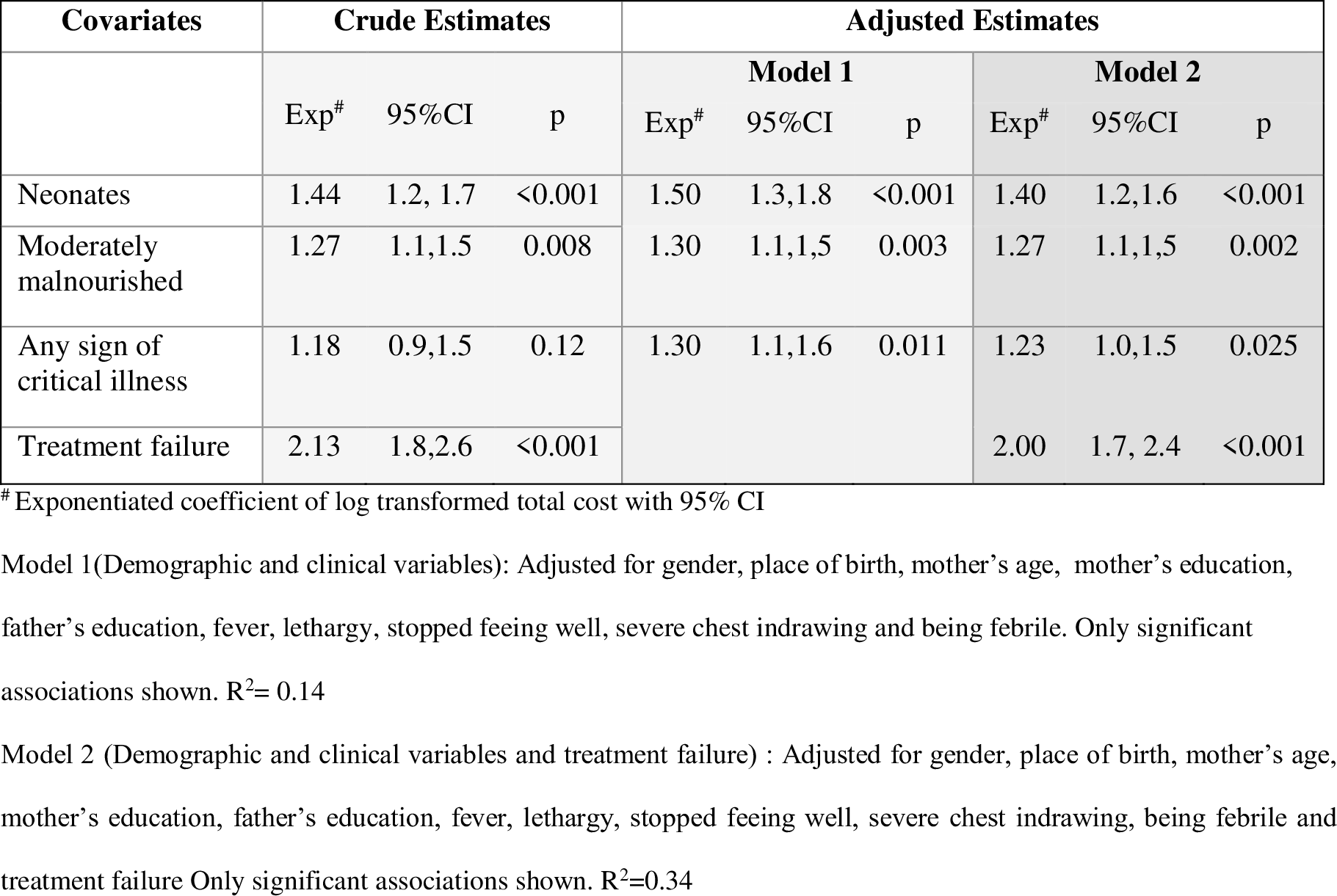

Supplement: S2 Table — (TIF) [file pone.0260127.s002.tif]

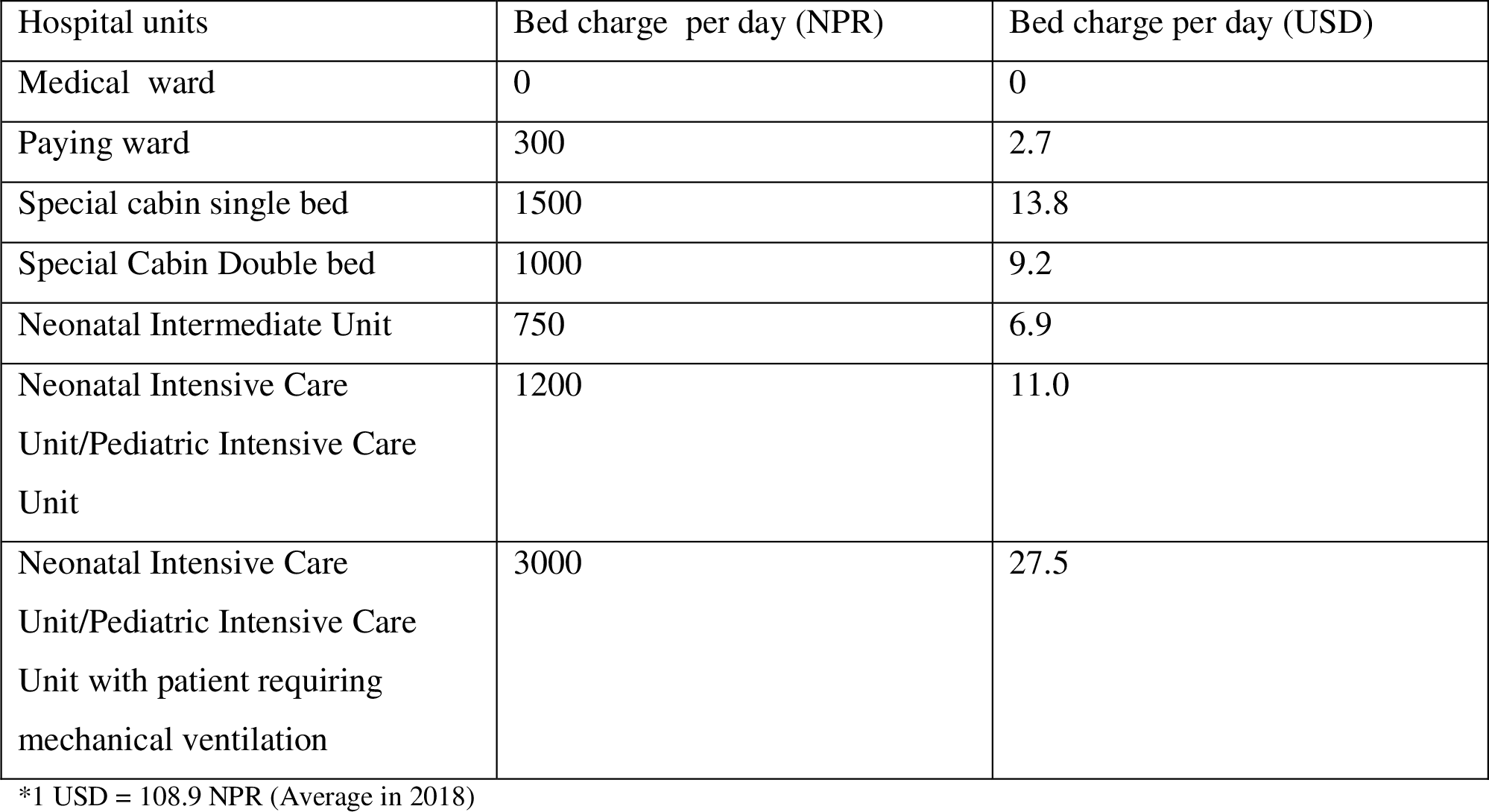

Supplement: S3 Table — (TIF) [file pone.0260127.s003.tif]
