# Supplementary material for: Assessment of hospitalization costs and its determinants in infants with clinical severe infection at a public tertiary hospital in Nepal
Source: PLoS One. 2021 Nov 29;16(11):e0260127. doi: 10.1371/journal.pone.0260127 (PMC8629207; doi:10.1371/journal.pone.0260127)
Supplement: Attachment — Submitted filename: Response to reviewers.docx [file pone.0260127.s005.docx]

1. Based on previous comments to enhance clarity on sepsis and clinical severe infection, the authors may need to add more details for relation between sepsis and clinical severe infection as the abstract starts with sepsis (line 26) and in the aims of the study mentions estimation of costs of clinical severe infection (line 30). Same holds for first para in introduction.

- **Thank you for your suggestion. We have added a few lines in the abstract (Lines 30 to 31) and also to the introduction section (Lines 60 to 70) explaining this relationship and hope that it is clear now . This required the addition of 4 extra citations that have been added to the reference section.**

2. The title should include public sector tertiary hospital if this was the case as mentioned in line 44.

- **We have now added the suggested, ‘public’ in the title**

3. Lines 121 to 123 mention about various cost centres that are directly involved in provision of care to infants/neonates. It would be useful to have granular details of unit cost by these cost centres in the supplement file since 74% of the recruited patients were in paying wards and private cabins.
**We are unable to provide granular details of unit cost, disaggregated by the ‘cost centres’. However, we have prepared a supplementary table (S3 Table) with details of cost per day of each cost centre.**

**We would also like to clarify that 74% admitted to other wards (private and paying) represents 50 out of 68 young infants and not all enrolled study participants. The remaining (138 out of 206) comprised of neonates admitted to Neonatal Intermediate wards and a Neonatal Intensive Care unit.**

4. Line 160-161: It would be useful to know the average length of stay in different cost centres-wards, paying wards, private cabins, ICU settings for reimbursement rates purpose to arrive at per bed day cost in these settings. This could be added in the supplement file.

**-Thank you for your suggestion. We agree that it would be useful to know the average length of stay in different cost centres for reimbursement purpose to arrive at per bed day cost. As stated in the earlier response we are unable to provide this data.**

5. Line 266 could be rephrased to direct medical costs as authors did not collect information of direct non-medical costs.

**- Thank you for pointing this out. We have rephrased as suggested.**

6. Supplementary table S3 with geometric means can be excluded. This is too much information for readers which will confuse them. Moreover, authors have already provided median estimates in the main manuscript and I assume median and GM to be close.

- **We have now removed geometric mean from the Supplementary table**

7. Table 2, page 8: I suggest the 4 and 5 digits median cost estimates should not be shown in decimals. It is better if the cost estimates upto 3 digits are shown in decimals (that also only upto one decimal place). Same suggested for figure 2. Also, give the full form of IQR under the table 2.

- **We have now revised the number of decimals as suggested**

8. The font size of text varies at several places such as reference 20 and; page 3 and 4. Also, there seems to be inconsistency in text spacing. The manuscript should be read carefully to check the same.

**- We have now corrected the font size in the manuscript and modified the spacing according to requirements of the journal.**
